# Translation, validation, and comparison of genetic knowledge scales in Greek and German

**DOI:** 10.3389/fgene.2024.1350308

**Published:** 2024-05-15

**Authors:** Florian Melchior, Konrad Beyreuther, Birgit Teichmann

**Affiliations:** Network Aging Research (NAR), Heidelberg University, Heidelberg, Germany

**Keywords:** genetics, genetic testing, knowledge, gender, autonomy, religiosity

## Abstract

**Introduction** **:** Advances in biosciences have significantly expanded our knowledge and capabilities in medicine and technology. Genetic tests can now predict hereditary predisposition or susceptibility to diseases, while gene-editing tools like CRISPR/Cas enable easy repair of disease genes in both somatic and germline cells, ensuring permanent genome correction. Despite these advancements, there is a shortage of valid instruments for studying the knowledge about these technologies. To fill this gap, our study aims to translate and validate various scales to effectively measure the public’s knowledge of genetics.

**Methods:** A convenience sample of *N* = 567 (Germany *n* = 317, Greece *n* = 250) participants completed a Google Forms questionnaire between December 2022 and June 2023, which included the General Knowledge of Genes and Heredity (GKGH), Knowledge about Gene-Environment Interaction (KGEI), and Knowledge of Modern Genetics and Genomics (KMGG) questionnaires. Analyses included internal consistency, structural validity, construct validity, and retest reliability with a subset of *n* = 72 (DE) and *n* = 50 (GR). Correlation analyses and group differences were evaluated for gender, education, religiosity, age, prior experience with genetic testing, and preferences toward potential providers of genetic testing. This study used the STROBE checklist for reporting.

**Results:** The GKGH exhibited low values in internal consistency and item analysis, along with a ceiling effect within the German group. However, it demonstrated good values in retest and construct validity. In the Greek group, all properties were highly satisfactory. The KMGG consistently displayed excellent properties across all analyses, whereas the KGEI only showed convincing results in construct validity and item analysis.

**Discussion:** The GKGH and KMGG demonstrated strong psychometric properties with varying difficulty levels dependent on the sample, with the German sample demonstrating a notably higher understanding of genetic technologies. Despite displaying acceptable properties, the KGEI fell short of measuring what its title suggests. Participants’ level of education showed a significant correlation with knowledge of genetic technologies, and only in the Greek sample did experiences with genetic tests influence knowledge. Preferences regarding availability of genetic testing are comparable between the two countries, with variations influenced by factors such as age, gender and religiosity.

## Introduction

In recent decades, the development of modern life sciences has expanded our ability to know and act in medicine and technology to an unprecedented degree. One of the areas that has become particularly important is genomics. The genomic era began with the sequencing of the human genome in the Human Genome Project (Collins et al., 2003), which made the genetic tests possible and enabled the prediction of “genetic predisposition” – an inherited tendency or susceptibility to certain diseases. The new “possibilities” of predicting disease before symptoms appear, even to the point of intervening in genes to modify them accordingly, may improve quality of life but may also raise ethical questions. Despite the attention of the media, it is to question whether the hype surrounding genomics is falling short of expectations in terms of prevention, diagnosis, and treatment (Henneman et al., 2013).

Through the discovery of new genes in recent years, an increasing number of genetic tests for specific diseases have been developed. Since the early 2000s, it has been possible to bypass healthcare professionals through the use of direct-to-consumer (DTC) genetic testing (Kalokairinou et al., 2018), as more companies offer a wide range of genetic tests. In addition to often lacking clinical validity and utility, the results of such tests are complex and difficult to interpret without additional information, such as accurate pedigree analysis. Therefore, it is important to question their usefulness, as harmful health decisions may result from misconceptions and misinterpretations (Phillips, 2016). In addition, data protection issues arise because neither informed consent nor the identity of the subjects can be verified beyond doubt, and because genetic data are considered sensitive data under the GPDR, for which processing is subject to special standards (Mondschein and Monda, 2019).

The use of genetic testing is governed by national laws, although efforts are underway at the European and international levels to establish overarching regulations for DTC testing, as well as a common accreditation system for genetic counselors. Currently, there is a wide variation in the number, role, and training of genetic counselors across Europe (Cordier et al., 2012).

In Germany, the requirements for qualification and the content of genetic counseling are established by the guidelines of the Genetic Diagnosis Commission (GEKO, 2011). According to the Genetic Diagnosis Act, “diagnostic genetic testing may only be performed by physicians, and predictive genetic testing may only be performed by specialists in human genetics or other physicians who have qualified in their respective medical field by obtaining a specialist, focus, or additional qualification for genetic testing” (Bundesministerium für Gesundheit, 2016).

Since the wording in the Oviedo Convention is relatively vague, applying to all European states that have signed and ratified it (“For predictive genetic tests as referred to in Article 12 of the Convention on Human Rights and Biomedicine, appropriate genetic counselling shall also be available for the person concerned”–(Conseil de l'Europe, 1997), some countries, such as Greece, do not have corresponding training programs for genetic counselors, nor do they have a professional association or recognition of genetic counseling in Greek legislation (Fountzilas et al., 2022).

The fundamental requirement to determine when a genetic test is appropriate and what type of interpretation it allows is a sufficient understanding of the genetic component of the disease (Cappelli et al., 1999). In the past, several population studies have been conducted to examine knowledge and attitudes toward genetics, genomics, and genetic testing. In these studies, both validated questionnaires (such as GKGH) (Jallinoja and Aro, 1999) and custom questionnaires were employed (Etchegary et al., 2010).

To capture psychological constructs such as knowledge about a topic, reliable and valid measurement tools are needed, and currently, there is no such tool available for measuring genetic knowledge in German and Greek languages. A widely used questionnaire is the “General Knowledge of Genes and Heredity” (GKGH). It was originally developed by Jallinoja and Arja (Jallinoja and Aro, 1999) and subsequently used to examine genetic knowledge in the general population (Jallinoja and Aro, 2000; Haga et al., 2013) in patients with chronic diseases (Calsbeek et al., 2007) as well as to evaluate the difference between healthy people and patients with immune diseases (Khdair et al., 2021). The questionnaire contains 16 structured items related to scientific facts about DNA, genes, cells as well as questions regarding the association of genes with a disease.

Carver et al. (2017) have developed a more extensive questionnaire called “Public Understanding and Attitudes towards Genetics and Genomics (PUGGS),” which is designed to investigate beliefs in genetic determinism, knowledge of modern genetics as well as attitudes toward modern genetics and genomic-based technologies. The PUGGS item sets were developed for use individually or together in order to facilitate the exploration of possible relationships among constructs (Carver et al., 2017). Furthermore, since it is a more recent questionnaire, it contains items related to epigenetics, which did not exist during the development of the GKGH. While Carver et al. (2017) suggested that the sections “Knowledge about Gene-Environment Interaction” and “Knowledge about Modern Genetics and Genomics” form a scale, Tornabene et al. (2020) argued that they should be considered as two parallel questionnaires and require additional validation.

Due to the lack of an established, validated tool for assessing knowledge in genetic technologies, and considering that these three questionnaires cover slightly different subject areas, the primary goal of this study is to translate them into German and Greek, validate them in their corresponding general population and perform a comparative analysis of the three questionnaires, regarding socioeconomic factors such as age, gender, religion, and education. This will allow for an evaluation of the psychometric properties and provide recommendations for future projects. In addition, we employed a concise survey developed by Chokoshvili et al. (2017) to assess attitudes toward providers of genetic testing services. This survey aims to offer insights into the preferences for the availability of genetic testing in both countries.

## Materials and methods

A convenience sample was recruited between December 2022 and May 2023 in Germany and between January 2023 and June 2023 in Greece through flyers and newsletters and by forwarding the call to participate in the study via social media such as WhatsApp and Facebook.

The final sample size was *N* = 317 for the German (DE) group and *N* = 250 for the Greek (GR) group. After 4 weeks, some of the participants completed the questionnaire a second time. This resulted in a subsample of *n* = 72 for the German sample and *n* = 50 for the Greek.

The present study followed the EQUATOR guidelines for reporting research using the “Strengthening the Reporting of Observational Studies in Epidemiology” (STROBE) checklist (Elm et al., 2007) (Supplementary Material 1).

### Questionnaire design

The questionnaires discussed in this article were part of a larger project that included additional questionnaires about the moral judgment of genetic technologies (Melchior et al., 2024, Teichmann et al., 2024, both under review). Google Forms were used to collect the data, which required participants to answer sequential questions about sociodemographic information, religiosity, previous experience with genetic testing, general questions about genetics {15 questions, adapted from Jallinoja and Aro (1999), gene-environment interactions [nine questions, adapted from Carver et al. (2017)]}, Knowledge about Modern Genetics and Genomics [16 questions, adapted from Carver et al. (2017)] and Potential Providers of Genetic Testing [adapted from (Chokoshvili et al., 2017)]. At the end of the first questionnaire, respondents were given the option to participate a second time after 4 weeks. They could create a code to match the data from two surveys and provide their email addresses to receive a reminder to participate the second time. The time needed to complete the questionnaire was estimated to be around 10–20 min.

### General Knowledge of Genes and Heredity (GKGH)

The GKGH questionnaire, originally published by Jallinoja and Aro (1999) measures the knowledge about the association of genes and diseases as well as the association of genes, chromosomes, cells, and the body. The original questionnaire originally consisted of 16 items, and Jallinoja and Aro were able to demonstrate a Cronbach’s alpha of 0.86. To precise the questions, the questionnaire was slightly modified. The statements “Gene is a molecule that controls hereditary characteristics” and “A gene is a piece of DNA” were removed because they posed nonspecific questions. Instead, the item “A gene is a section within DNA that contains the blueprint for a protein” was added. Furthermore, the item “It has been estimated that a person has about 70.000 genes”, which was previously adapted by others to 22.000 (Calsbeek et al., 2007), was changed to 20.000. These adjustments were made to reflect the current scientific state, as this statement was considered correct at the time of questionnaire development. Accordingly, this questionnaire consists of 15 statements, and for each question, respondents/participants can choose to answer with either “*True*,” “*False*,” or “*I don’t know*,” resulting in a score ranging from 0 to 15, whereby a higher score is associated with better knowledge about genetics.

### Knowledge about Gene-Environment Interaction (KGEI)

The KGEI, developed by Carver et al. (2017), is a component of the PUGGS questionnaire. It consists of nine statements regarding the correlation between genetic factors, environmental influences, phenotypes, and disease development. Respondents can answer these statements with “*True*,” “*False*,” or “*I don’t know*,” resulting in a scale ranging from 0 to 9 points. A higher score indicates a more comprehensive grasp of genomic-environmental interactions. In the pilot study, this version of the questionnaire demonstrated a Cronbach’s alpha coefficient of 0.67.

### Knowledge about Modern Genetics and Genomics (KMGG)

The KMGG is a part of the “Public Understanding and Attitudes towards Genetics and Genomics” questionnaire developed by Carver et al. (2017). It measures an individual’s objective knowledge of genetics and genomics. The KMGG focuses on three areas: (1) characteristics of the genome, (2) gene function and expression, and (3) epigenetics. It consists of 16 statements that can be answered with “*True*,” “*False*,” or “*I don’t know*,” The total score ranges from 0 to 16 points, with higher scores associated with better knowledge of genetics and genomics. The questionnaire achieved a Cronbach’s alpha of 0.69 and 0.70 in the pilot study.

### Preferences for Potential Providers of Genetic Testing

The “Preferences for Potential Providers of Genetic Testing” is a short questionnaire consisting of four individual questions regarding a person’s stance on the availability of genetic tests (Chokoshvili et al., 2017). An example question is: “Genetic tests may only be performed on a doctor’s prescription.” Respondents can answer these items on a five-point scale ranging from “strongly disagree” to “strongly agree;” however, this scale does not represent a latent construct.

### Developing the German and Greek version of the questionnaires

The translation back-translation method (Hambleton, 2001) was used to translate the English version of the questionnaire into German and Greek. Specifically, two native speakers separately translated the original English version into German and Greek, respectively. Differences in translation were discussed with the research team to ensure cultural adaptation, and a synthesis of the two translations was produced. The Greek and German versions were back-translated by two people each, who were either native speakers or translators. The original English version and the back-translated versions were compared for consistency, relevance, and meaning of the content. The final version was administered to three researchers with expertise in genetics to ensure that all items were consistent before the questionnaires were finalized. The translated questionnaires are included in the Supplementary Table S2.

### Statistical analysis

The data was analyzed using descriptive and inferential statistical methods with IBM SPSS Statistics Version 27 (IBM Corp, 2022). The psychometric properties of all questionnaires measuring a psychological construct were evaluated, including internal consistency (Cronbach’s alpha), structural validity (Principal Component Analysis, PCA), construct validity (known-groups method), item analysis, floor and ceiling effects, and retest reliability. The “Preferences for Potential Providers of Genetic Testing” questionnaires did not measure a psychological construct but instead proposed a series of isolated statements. Accordingly, we tested gender, religiosity, education, and age groups for differences.

### Cronbach’s alpha and retest reliability

To ensure the internal consistency of our measurements, we computed Cronbach’s alpha, a metric that gauges the degree of shared variance among items. The generally accepted range for Cronbach’s alpha, as recommended, falls between 0.70 and 0.90 (Tavakol and Dennick, 2011).

To evaluate the test-retest reliability, we compared data from the entire sample (*N* = 317 for DE and *N* = 250 for GR) with a subsample (*n* = 72 for DE and *n* = 50 for GR) after a 4-week interval. We then calculated the interclass correlation coefficient, which quantifies the similarity between the two sets of surveys. To determine retest reliability, we followed the method outlined by Koo and Li (Koo and Li, 2016) in SPSS. This method employs a two-way mixed effects model, considering the mean of k measurements and absolute agreement.

Unfortunately, due to the inclusion of incorrect codes, we were unable to identify three individuals, and thus, they were excluded from the retest analysis. Consequently, the retest was conducted with a total of 69 participants for the German group and 50 for the Greek group.

### Construct validity

To evaluate construct validity, we employed the known-groups method, a technique that distinguishes two groups based on anticipated differences in their scale scores. The study used a self-report variable ranging from 1 to 7 to measure self-assessed genetic knowledge. The sample was then divided into a “l*ow*” knowledge group (those scoring 1 or 2) and a “*high*” knowledge group (those scoring 6 or 7). We hypothesized that individuals in the high-knowledge group would demonstrate superior performance on tests evaluating their understanding of genetic technologies. Consequently, we formulated the following hypothesis: (1) The group self-assessed as highly knowledgeable in genetic technologies will attain a higher overall score on the questionnaire. To assess this hypothesis, we employed the Wilcoxon-Mann-Whitney test (WMW) (Mann and Whitney, 1947) for all the questionnaires.

### Power analysis

To ensure sufficient statistical power for our analyses, we conducted a power analysis using G*Power 3.1.9.7 software (Faul et al., 2007) following Kang’s guidelines (2021) (Kang, 2021). For the hypothesis mentioned earlier, we anticipated an effect size of at least *d* = 0.5, aiming for a desired power of 1 − β = 0.95 and maintaining a significance level of α = 0.05.

Given our prior studies (Teichmann et al., 2022; Melchior and Teichmann, 2023), which indicated a slightly over-educated sample due to our recruitment strategy, we adjusted the allocation ratio to 3. Consequently, we expected a higher proportion of individuals with advanced knowledge of genetic technologies in our sample, necessitating a larger sample size. Based on G*Power’s recommendation, we arrived at a total sample size of *N* = 244 for the Wilcoxon-Mann-Whitney tests.

### Item analysis

We performed an item analysis for each questionnaire to evaluate the item-total correlation for all the items. This correlation measures how consistent an individual item’s score is with the overall scale score, offering valuable insights into the contribution of each item to the measurement. Additionally, we examined the inter-item correlation to gauge the strength of relationships between different items.

Typically, item-total correlations and mean inter-item correlations in the range of 0.2–0.4 are seen as indicative of significant informational contributions to the scale. It is worth noting, however, that higher correlations do not necessarily imply increased reliability. In fact, excessively high correlations may signal item redundancy, which can artificially inflate the questionnaire’s internal consistency (Ferketich, 1991; Rattray and Jones, 2007; Piedmont, 2014).

### Floor and ceiling effects

Another important aspect to take into account is the potential presence of ceiling or floor effects. Ceiling and floor effects occur when observations cluster at the maximum or minimum values, respectively, such as achieving a perfect score. As a result, data accumulating at these extreme values creates a ceiling or floor effect, which can distort the data distribution and introduce bias. This bias can lead to misleading results, especially in analyses that assume a normal distribution (Šimkovic and Träuble, 2019).

Although specific thresholds for identifying these effects are not universally standardized, we considered a ceiling or floor effect to be present if more than 10% of all participants scored at the minimum or maximum level on any questionnaire. Furthermore, we conducted a specific re-evaluation of ceiling effects within the sample possessing advanced knowledge of genetic technologies to assess whether the presence of a ceiling effect was contingent on the characteristics of that sample.

There was no missing data, as Google Forms only accepted completed records.

### Ethical considerations

This study was approved by the Ethics Committee of the Faculty of Behavioral and Empirical Cultural Sciences of Heidelberg University (AZ Teich 2022 3/1) and by the Scientific and Ethics Committee of the Greek Association of Alzheimer’s Disease and Related Disorders (GAADRD, Approved Meeting Number: 084/18-01-2023). All procedures involved in this work conformed to the ethical standards of the Declaration of Helsinki, as applicable to national and institutional human experimentation committees. Prior to the survey, written informed consent was obtained from each participant, and they were informed that the research was voluntary, confidential, and for academic purposes only. Email addresses were removed after merging data from different time points to ensure anonymity.

## Results

### Sociodemographic data

A total of 567 people participated in the study: 317 in the German sample and 250 in the Greek sample. Table 1 shows the sociodemographic data for the two complete samples. The average age was 43.53 years for the DE sample and 41.20 years for the GR group, with most participants being female (DE: 69.7% and GR: 64.4%). The most common level of education attained is a master’s degree or diploma, with 38.8% in the DE sample and 42.0% in the GR sample and with most participants in the German group working in academic (28.1%) and in “others” in the Greek sample (32.0%).

**TABLE 1:** Participants’ characteristics of the German and Greek sample.

Characteristics	German sample (*N* = 317)		Greek sample (*N =* 250)	
	*n*	%	*n*	%
Age				
Mean	43.53		41.20	
SD	17.87		13.16	
Gender				
Male	93	23.3%	89	35.6%
Female	221	69.7%	161	64.4%
Diverse	3	0.9%	0	0%
Education				
9 years or less	0	0	5	2.0%
10 years	7	2.2%	1	0.4%
12–13 years	70	22.1%	17	6.8%
Vocational training	35	11.0%	32	12.8%
Bachelor	36	11.4%	56	22.4%
Master/Diploma	123	38.8%	105	42.0%
PhD	44	13.9%	34	13.6%
Others	2	0.6%	0	0%
Occupation				
School student	2	0.6%	0	0%
Student	78	24.6%	22	8.8%
Unemployed	3	0.9%	17	6.8%
Retiree	37	11.7%	20	8.0%
Care profession	11	3.5%	16	6.4%
Therapeutical profession	21	6.6%	29	11.6%
Physician	12	3.8%	15	6.0%
Academic	100	28.1%	51	20.4%
Others	53	20.2%	80	32.0%
Marital status				
Divorced	20	6.3%	16	6.4%
In partnership	81	25.6%	55	22.0%
Single	84	26.5%	65	26.0%
Married	126	39.7%	113	45.2%
Widowed or deceased partner	6	1.9%	1	0.4%
Do you have children?				
Yes	142	44.8%	113	45.2%
No	175	55.2%	137	54.8%
Have you ever had a genetic test done?				
Yes	20	6.3%	26	10.4%
No	297	93.7%	224	89.6%
Has genetic testing ever been performed on a close friend or relative?				
Yes	72	22.7%	60	24.0%
No	128	40.4%	83	33.2%
I don’t know	117	36.9%	107	42.8%
Would you like to have a genetic test performed?				
Yes	78	24.6%	109	43.6%
No	103	32.5%	40	16.0%
I don´t know	136	42.9%	101	40.4%
Self-assessment of knowledge about genetic technologies				
Low (1–2)	36	11.4%	66	26.4%
Medium (3–5)	140	44.2%	107	42.8%
High (6–7)	141	44.5%	77	30.8%

The survey revealed that when asked about genetic testing, 6.3% of Germans and 10.4% of Greeks have undergone genetic testing, and 22.7% of Germans and 24.0% of Greeks have had genetic testing performed on a close friend or relative. While 24.6% of the DE sample and 43.6% of the GR sample are interested in undergoing a genetic test, 32.5% of Germans and 16.0% of Greeks have no interest in the test.

Religiosity was assessed by three questions about self-rated religiosity, frequency of attending religious services, and how religion influences decisions, which were summed. The mean was M = 5.79 (SD = 2.77) on a scale from 3 to 15 for the German group and M = 7.41 (SD = 3.14) for the Greek group. In our analysis of the two samples, we divided the variable for the level of the religiosity into three categories: low (<5), medium (6–10), and high (>10). Our results indicate that most people (n = 176) of the DE sample have low religiosity, while 116 people have a medium level of religiosity, and only 25 people have a high religiosity. In the GR group, the majority of the participants had a medium level of religiosity (*n* = 133), followed by the “low” group (*n* = 75). The smallest group of n = 42 was considered “high.”

In the self-assessment of how much a participant knows about gene technologies compared to the general population, a mean value of M = 4.31 (SD = 1.38) was achieved on a scale of 1–7 for the German group and a mean of M = 3.66 (SD = 1.63) for the Greek group.

In Table 2, the correlation of the three knowledge questionnaires with the variables gender, age, years of education, religiosity, and self-assessed knowledge of genetic technologies is presented. Significant positive correlation with all questionnaires was achieved only by the length of education and self-assessed knowledge. Religiosity and age were significantly, albeit slightly, negatively correlated with two questionnaires, and the gender of the participants did not correlate with any of the three scales.

**TABLE 2:** Correlation between the variables for the total sample.

	Gender	Age	Years education	Religiosity	Self-assessed knowledge
GKGH	0.050	−0.046	0.271**	−0.109**	0.525**
KGEI	0.053	−0.149**	0.189**	−0.105*	0.370**
KMGG	0.036	−0.098*	0.209**	−0.081	0.494**

*N* = 567. *p < 0.05; **p < 0.01.

### General Knowledge of Genes and Heredity

#### Descriptive statistics

The GKGH questionnaire had a mean score of 12.41 (SD = 2.10) on a scale from 0 to 15 in the German group, whereas the mean for the Greek group was M = 9.93 (SD = 3.33).

The questionnaire was answered correctly, with an average of 82.8% in the German sample, while “*I don’t know*” responses were given for approximately 12.2% of the questions. In the Greek group, the percentage of correct answers averaged 66.2%, while 27.0% were “*I don’t know*” responses.

#### Cronbach’s alpha and retest reliability

The values for internal consistency can be found for all questionnaires in Table 3, where Cronbach’s alpha is reported separately for the total sample and for the group with low self-assessed knowledge and high knowledge. The results of the retest are presented for all questionnaires in Table 4.

**TABLE 3:** Psychometric properties of all tested questionnaires.

	Mean score (SD)	Cronbach’s alpha			Mean item-total correlation	Mean inter-item correlation	Correct (%)	Don’t know (%)
GKGH^a^	*Range: 0–15*	*Total sample*	*LK* ^b^	*HK* ^c^				
Germany	12.41 (2.02)	0.646	0.654	0.446	0.267	0.109	82.8	12.2
Greece	9.93 (3.33)	0.814	0.787	0.789	0.422	0.219	66.2	27.0
KGEI^d^	*Range 0–9*							
Germany	6.58 (2.24)	0.759	0.809	0.572	0.437	0.257	73.3	10.4
Greece	4.80 (2.56)	0.784	0.760	0.711	0.469	0.286	53.1	27.2
KMGG^e^	*Range 0–16*							
Germany	8.67 (4.20)	0.852	0.836	0.833	0.436	0.264	54.2	23.4
Greece	5.37 (3.89)	0.840	0.788	0.843	0.457	0.251	33.5	41.2

^a^ General Knowledge of Genes and Heredity questionnaire.

^b^ LK = Low self-assessed knowledge about genetic technologies.

^c^ HK = High self-assessed knowledge about genetic technologies.

^d^ Knowledge about Gene-Environment Interaction questionnaire.

^e^ Knowledge of Modern Genetics and Genomics questionnaire.

**TABLE 4:** Test-retest reliability with the subgroups.

	Intraclass Correlation Coefficient	95%—Confidence interval	
		Lower bound	Upper bound
GKGH			
DE	0.851	0.759	0.908
GR	0.935	0.883	0.964
KGEI			
DE	0.664	0.461	0.791
GR	0.797	0.635	0.887
KMGG			
DE	0.851	0.758	0.908
GR	0.800	0.638	0.889

Retest groups were 69 participants for the German sample and 50 for the Greek sample.

For the DE sample, the GKGH was able to achieve a Cronbach’s alpha of 0.646. However, in the group with a higher level of knowledge about genetic technologies, the value was only 0.446. The GR group had an alpha value of 0.814 for the total sample. In contrast to the DE group, the significant decline in internal consistency for the high-knowledge group was absent in the Greek sample. The two high-knowledge groups had a comparable mean score: The DE group had a mean of M = 12.30 (SD = 1.40), and the GR group had a mean of M = 12.23 (SD = 2.65). However, an important difference between the groups was the item difficulty for the individual items. While the Greek sample only had two items with >95% correct answers, the German group achieved >95% correct answers in eight different items and even 100% correct answers in item 2. On the other hand, the retest was satisfactory, with a confidence interval between 0.759 and 0.908 for the German sample and 0.883 to 0.964 for the Greek.

#### Construct validity

The known-groups method for the GKGH as well as for all other questionnaires is included in Table 5. In both the German (z = 5.157; *p* < 0.001) and Greek sample (z = 7.076, *p* < 0.001), the GKGH successfully distinguished between the low- and high-knowledge groups.

**TABLE 5:** Results of the Wilcoxon-Mann-Whitney tests for all questionnaires.

Questionnaire	Mean rank		U^c^	z^d^	*p* ^e^
	Low knowledge^a^	High knowledge^b^			
GKGH					
DE	50.63	98.8	3,919.5	5.157	<0.001
GR	45.65	94.58	4,280.0	7.076	<0.001
KGEI					
DE	51.88	98.48	3,874.5	4.991	<0.001
GR	55.98	85.73	3,598.0	4.313	<0.001
KMGG					
DE	41.94	101.01	4,232	6.190	<0.001
GR	51.44	89.62	3,898.0	5.518	<0.001

^a^ Low self-assessed knowledge about genetic technologies: *n* = 36 in the German group and *n* = 66 in the Greek group.

^b^ High self-assessed knowledge about genetic technologies: *n* = 141 in the German group and *n* = 77 in the Greek group.

^c^ U = U test statistic.

^d^ z = z statistic.

^e^ *p* = significance.

#### Item analysis and floor and ceiling effects

The item analysis revealed some significant differences in GKGH between the German and Greek samples, and the data are included in Table 3. While the German sample achieved a good mean item-total correlation of 0.267, the mean inter-item correlation fell below the desired range with a value of 0.109. These issues were not present in the Greek group. Here, the questionnaire achieved a mean item-total correlation of 0.422 and a mean inter-item correlation of 0.219, both of which were within the desired range.

**FIGURE 1:**
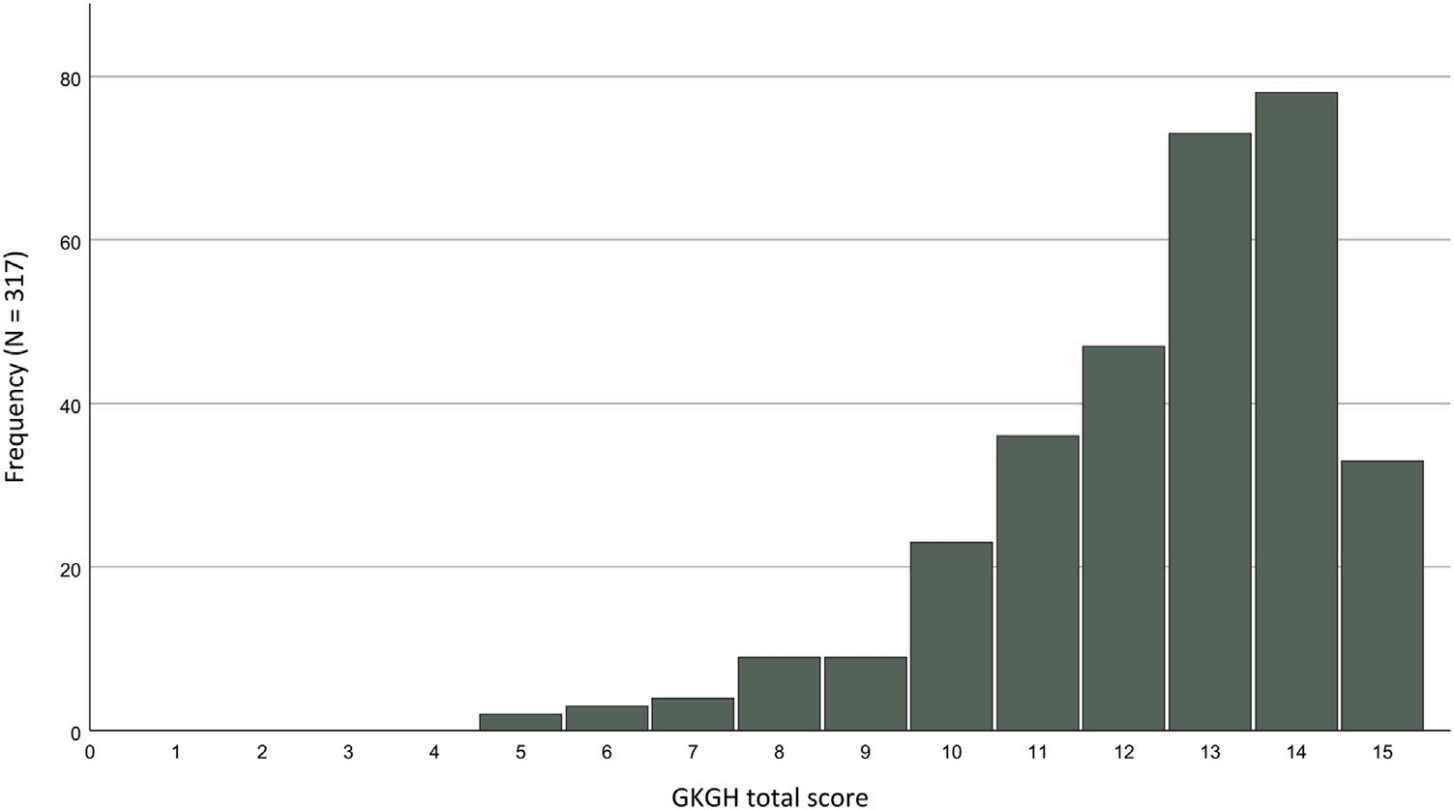
Ceiling effect of the GKGH total scores for the German sample.

Considering the distributions of the two groups, it becomes evident that there is a significant ceiling effect in the German sample, as 10.4% of the participants reached a maximum score of 15 points. This effect is displayed in Figure 1. However, this effect is not present in the Greek group, where only 6.0% of individuals were able to achieve a maximum score.

#### Structural validity

The Greek and German groups both achieved a sufficient KMO value (0.652 and 0.841), and both groups had a significant Bartlett test (*p* < 0.001). In the German group, three factors were extracted based on the Screeplot. These factors encompassed the following subjects: 1) general knowledge about genes (items 4, 8, 5, 6, 7, 1, 10), hereditary diseases (items 15, 14, 11), and a factor that included the remaining items but did not capture a consistent subject (items 2, 13, 3, 12). Item 9, “The genotype is not accessible for human interventions,” could not be assigned to any factor.

In the Greek group, the results were similar. Here, three factors were extracted as well, consisting of items related to general knowledge (items 8, 4, 5, 6, 7, 10), hereditary diseases (items 13, 12, 11, 14, 15), and a factor with the remaining items (items 1, 2, 3). Item 9 also could not be clearly assigned to a specific factor.

### Knowledge about Gene-Environment Interaction

#### Descriptive statistics

The KGEI had a mean of 6.58 (SD = 2.24) on a scale from 0 to 9 for the German sample and a mean of 4.80 (SD = 2.56) for the Greek sample.

In the German sample, the items of the KGEI were answered correctly on average at a rate of 73.3%, and “*I don’t know*” responses were chosen for an average of 10.4% of the questions. In the Greek sample, the items were answered correctly at a rate of 53.1%, and “*I don’t know*” responses were selected in 27.2% of cases.

#### Cronbach’s alpha and retest reliability

The scale achieved a satisfactory alpha of 0.759 and 0.784 for DE and GR, respectively. Nonetheless, the internal consistency noticeably declined in the German high-knowledge group to 0.572, while it remained relatively stable in the Greek group, with a value of *α* = 0.711. The retest revealed a rather low value for the German group, with a confidence interval between 0.461 and 0.791 and an acceptable interval between 0.635 and 0.887 for the Greek group.

#### Construct validity

The construct validity of the questionnaire was confirmed through the known-groups analysis for both groups, as it successfully differentiated between the low- and high-knowledge groups (DE: z = 4.991, *p* < 0.001; GR: z = 4.313, *p* < 0.001).

#### Item analysis and floor and ceiling effects

In the item analysis, the KGEI achieved good values for mean item-total and mean inter-item correlations in both the German and Greek samples. With mean item-total correlations of 0.437 for DE and 0.469 for GR, these values are slightly above the optimal range. The mean inter-item correlations, on the other hand, fall within the desired range with values of 0.257 and 0.286 for DE and GR, respectively.

**FIGURE 2:**
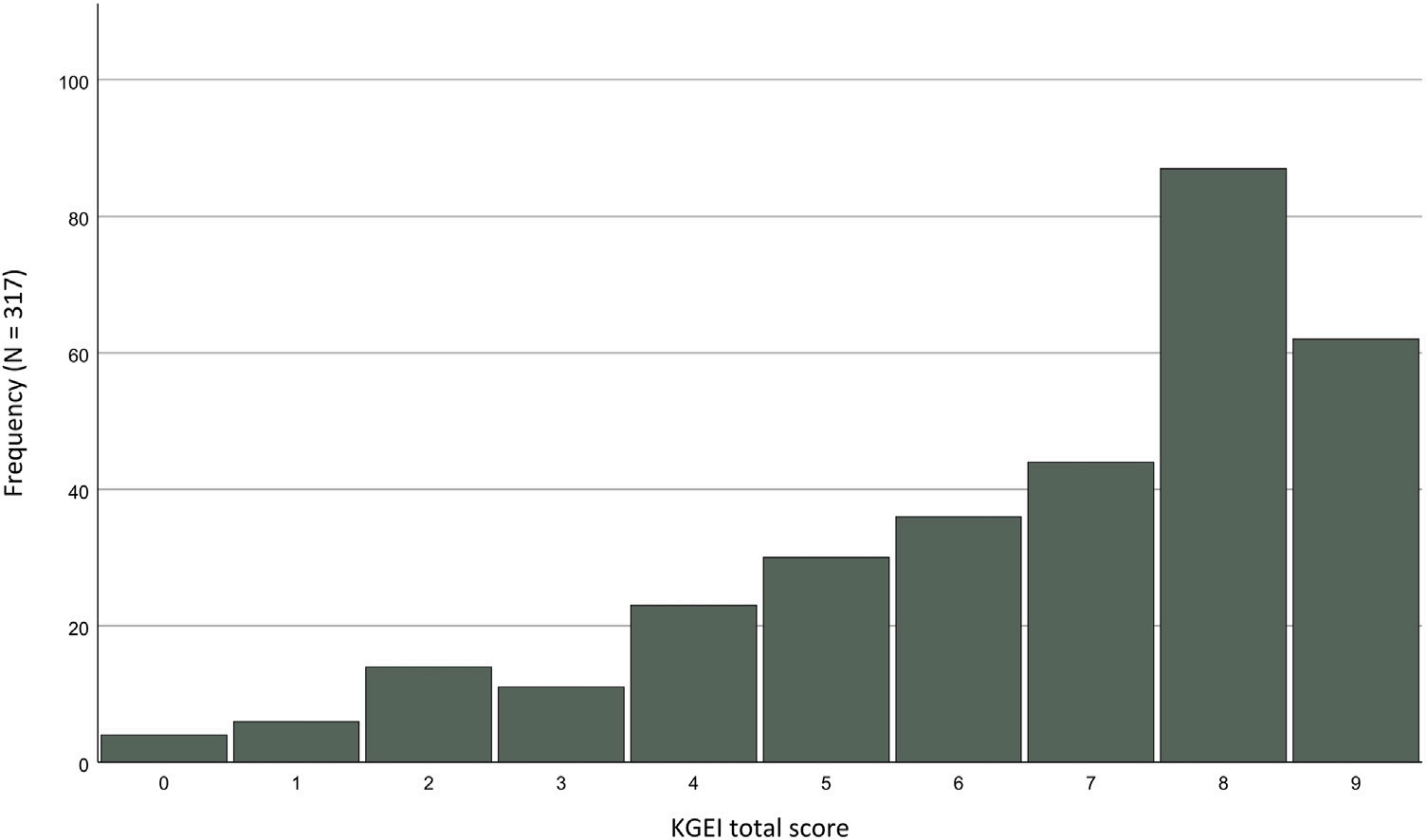
Ceiling effect of the KGEI total scores for the German sample.

However, the questionnaire suffers from a significant ceiling effect in the German group, which is evident in the fact that 19.6% of participants in the total sample achieved the maximum score of 9 points and clearly visible in the distribution diagram displayed in Figure 2. In the Greek sample, on the contrary, no ceiling or floor effects were observed; only 4.8% of participants achieved the maximum score, and 8.0% reached the minimum score.

#### Structural validity

The prerequisites for conducting PCA were satisfied in both groups, as indicated by a KMO measure of 0.788 and 0.792 as well as the significance of both Bartlett tests. Two distinct factors were identified. The content of these factors is consistent in both groups: One factor encompasses items related to the impact of genes on traits and diseases (items 1, 2, 3, 7), while the other factor comprises items concerning environmental influences (items 6 and 8). However, for the remaining items, the factor loadings in both samples lack coherence and are consequently not straightforward to interpret.

### Knowledge of Modern Genetics and Genomics

#### Descriptive statistics

For the KMGG, the mean score in the German group was M = 8.67 (SD = 4.20), while in the Greek group, it was 5.37 (SD = 3.89), on a scale ranging from 0 to 16 points.

For this questionnaire, the German and Greek groups answered the questions correctly at a rate of 54.2% and 33.5%, respectively. “*I don't know*” responses were selected by the German and Greek groups at rates of 23.4% and 41.2%, respectively.

#### Cronbach’s alpha and retest reliability

The internal consistency of the KMGG remained consistently high, with values ranging from 0.833 to 0.852 across the overall sample, the low-knowledge group, and the high-knowledge group. This consistency was observed in both the German and Greek groups. The only exception was the Greek low-knowledge group, which exhibited a slightly reduced Cronbach’s alpha of 0.788. Both groups displayed favorable retest values, with the DE group showing a confidence interval ranging from 0.758 to 0.908 and the GR group with an interval spanning from 0.638 to 0.889.

#### Construct validity

In the known-groups analysis, construct validity was confirmed. The KMGG was able to differentiate between the low-knowledge and high-knowledge groups in both the DE (z = 6.190, *p* < 0.001) and GR sample (z = 5.518, *p* < 0.001).

#### Item analysis and floor and ceiling effects

For the German group, the questionnaire performed exceptionally well, with a mean item-total correlation of 0.436 and a mean inter-item correlation of 0.264. The only problems identified were a low item-total correlation for item 5, with a value of 0.167, and a high inter-item correlation between item 7 and item 11, with a correlation coefficient of 0.73. This is not surprising, as items 7 and 11 contain the same statement but in reverse.

In the Greek group, the results were identical, with the difference that items 7 and 11 did not exhibit a correlation that was too high. With an item-total correlation of 0.457 and an inter-item correlation of 0.251, the questionnaire shows desirable values.

**FIGURE 3:**
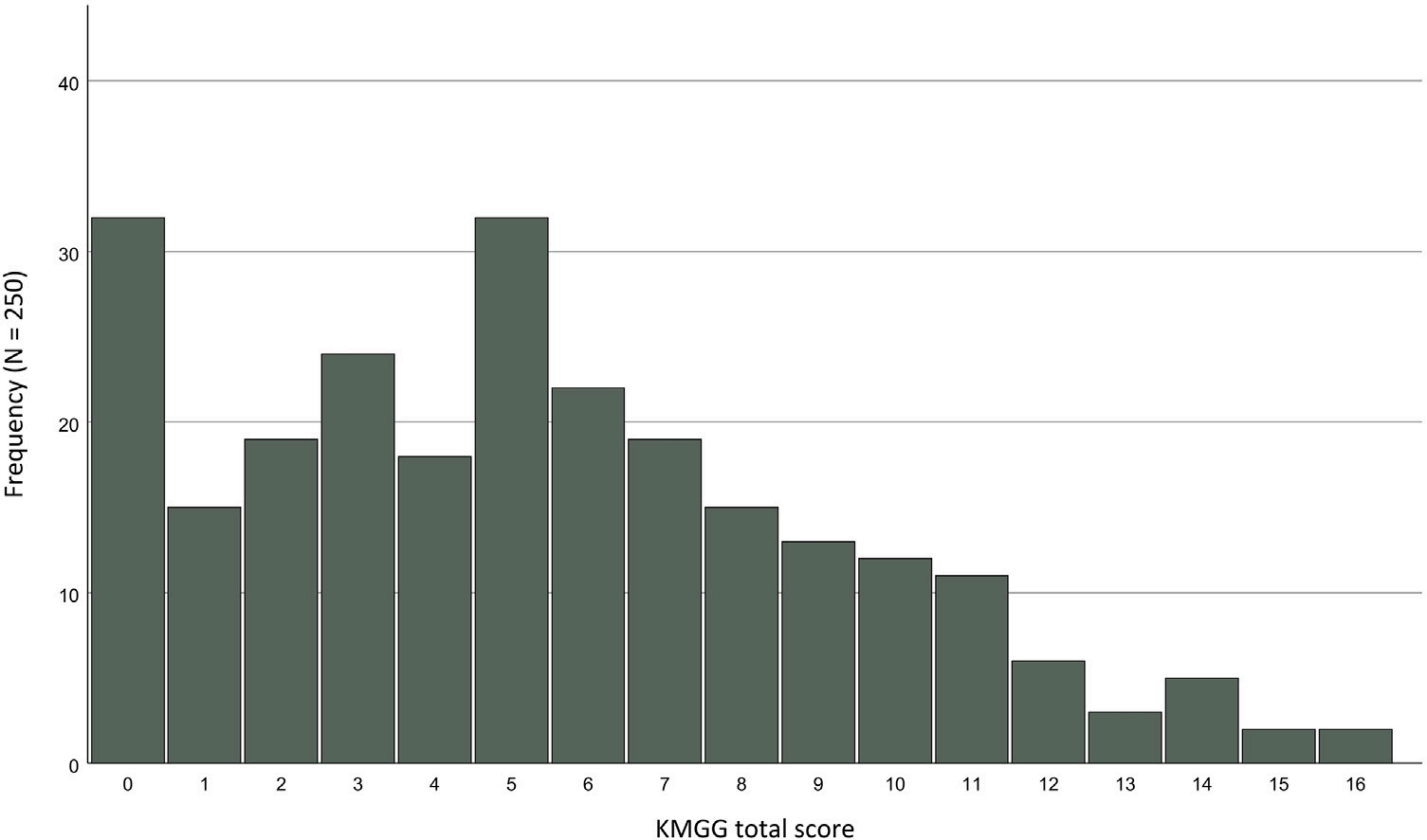
Floor effect of the KMGG total scores for the Greek sample.

When considering the distribution of scores, it is clearly noticeable that the Greek sample exhibits a significant floor effect, which is displayed in Figure 3. Whereas 12.8% of Greek participants achieved a score of 0 points, in the German group, it was 3.2%. At the same time, neither of the two groups displayed a ceiling effect.

#### Structural validity

The prerequisites have been met in both samples, with KMO values of 0.852 and 0.831 (for DE and GR) and significant Bartlett tests (*p* < 0.001). The German group exhibited three distinguishable factors: 1) general knowledge of genetics (items 15, 4, 9, 12, 10, 13, 5), statements about epigenetics (items 2, 8, 6, 16, 14, 3), and statements about protein production (items 7, 11, 1). In contrast, the Greek sample did not show clear factor distinctions, and the three factors are intermixed and not distinguishable from each other.

### Sociodemographic group differences

All three questionnaires were tested for differences in total scores for the variables age, gender, religiosity, and education. The tests were conducted for both the German and Greek groups. Table 6 displays the significant group differences of the Wilcoxon test.

**TABLE 6:** Sociodemographic differences for the three knowledge questionnaires.

	Age	Gender	Religion	Education
GKGH		GR*		DE**; GR**
KGEI	DE**	GR*		GR**
KMGG				DE**

Only significant results are displayed. DE, significant difference in the German group; GR, significant difference in the Greek group. *p < 0.05, **p < 0.01. Religiosity was split into low (<5) and high (>11). Age was split into low age (18–30 years) and high age (50+ years). Education was split into academic and non-academic.

The tests revealed that the age of the participants only showed a significant difference in the German group for the KGEI, where younger participants scored higher than older participants. Gender differences were only observed in the Greek group for GKGH and KGEI, with men scoring higher than women, and no significant group differences were detected between high and low religiosity. Significant differences in education were observed in all three questionnaires, though they were not present in all groups, with higher education consistently achieving higher scores.

By using Wilcoxon-Mann-Whitney tests, we also examined whether prior experiences with genetic testing had a significant impact on the knowledge scores in the questionnaires and whether the desire to undergo genetic testing influenced the knowledge score. The results are depicted in Table 7.

**TABLE 7:** Group differences for experience with genetic testing.

	Have you ever had a genetic test done?		*p*	Has genetic testing ever been performed on a close friend or relative?^a^		*p*	Would you like to have a genetic test performed?^b^		*p*
GKGH	*yes*	*no*		*yes*	*no*		*yes*	*no*	
DE	141.0	160.2	0.355	103.0	99.1	0.639	92.5	89.9	0.732
GR	166.6	120.7	**0.002**	79.4	66.6	0.066	74.7	75.9	0.878
KGEI									
DE	129.7	161.0	0.105	107.9	96.3	0.168	97.1	86.4	0.169
GR	147.1	123.0	0.132	83.2	63.9	**0.006**	75.4	73.9	0.851
KMGG									
DE	139.0	160.4	0.312	109.2	95.6	0.109	93.4	89.2	0.599
GR	157.3	121.8	**0.017**	80.3	66.0	**0.041**	75.2	74.6	0.942

^a and b^Only “*yes*” and “*no*” answers were included. Mean ranks are not comparable since they depend on sample size. Significant results are highlighted in bold.

No significant differences were observed in the German sample. However, in the Greek sample, participants who had undergone a genetic test had higher scores on the GKGH and KMGG questionnaires. In the case of the KGEI and KMGG questionnaires, participants who reported that a test had been conducted on a close friend or relative also achieved a significantly higher score.

### Preferences for Potential Providers of Genetic Testing

The results from the questionnaire on how genetic tests should be offered are included in Table 8.

**TABLE 8:** Preferences for potential providers of genetic testing.

	Strongly disagree	Disagree	Neutral	Agree	Strongly agree	Significant differences^a^	Group relation^b^
*N* (%)							
Item 1: Genetic tests may only be performed on doctor’s prescription							
DE	30 (9.5%)	70 (22.2%)	61 (19.3%)	101 (32.0%)	54 (17.0%)	Age**	High age > Low age
						Religion*	High rel. > Low rel
GR	17 (6.8%)	57 (22.8%)	52 (20.8%)	100 (40.0%)	24 (9.6%)	Age*	High age > Low age
						Religion*	High rel. > Low rel
Item 2: Genetic tests may only be performed in the hospital							
DE	44 (13.9%)	103 (32.6%)	83 (26.3%)	65 (20.6%)	21 (6.6%)	Gender*	Female > Male
GR	12 (4.8%)	88 (35.2%)	69 (27.6%)	62 (24.8%)	19 (7.6%)	none	
Item 3: Genetic tests may be sold through the Internet							
DE	150 (47.5%)	93 (29.4%)	41 (13.0%)	22 (7.0%)	10 (3.1%)	Age*	Low age > High age
						Religion*	Low rel. > High rel
GR	105 (42.0%)	78 (31.2%)	41 (16.4%)	23 (9.2%)	3 (1.2%)	Age** Gender*	Low age > High age
							Male > Female
Item 4: Genetic tests may be sold by a pharmacist							
DE	80 (25.3%)	89 (28.2%)	67 (21.2%)	60 (19.0%)	20 (6.3%)	Age**	Low age > High age
						Gender*	Male > Female
						Religion**	Low rel. > High rel
GR	67 (26.8%)	80 (32.0%)	64 (25.6%)	35 (14.0%)	4 (1.6%)	Gender**	Male > Female
						Religion**	Low rel. > High rel

^a^ In this column, only the significant group differences are presented.

^b^ This column expresses which of the two groups has a higher or lower agreement with the statement. DE sample size is 317, GR sample size is 250. Religiosity was split into low (<5) and high (>11). Age was split into low age (18–30 years) and high age (50+ years). *p < 0.05; **p < 0.01.

Approximately half of the respondents stated that genetic tests should only be conducted upon a doctor’s order, with older individuals and participants with high religiosity significantly more likely to agree with this statement. The statement that genetic tests should only be performed in hospitals was denied by most participants, and in the German group, female participants were more likely to agree with the statement than males. Only about 10% of participants in both groups agreed with the statement that genetic tests should be sold over the Internet, with younger individuals in both samples being more in favor of Internet sales than older individuals. The sale of genetic tests by pharmacies was rejected by most respondents, with younger, male, and non-religious participants in the German group being more inclined to agree with the statement. This tendency was also observed in the Greek group; however, age did not have a significant impact. Besides, there was no significant difference between academic and non-academic groups in any of the items.

## Discussion

The aim of the study was to translate and validate different scales regarding knowledge about genes, genetics, heredity, and gene-environment relationship in the German and Greek public. Because knowledge of genetics may play a major role not only in informed decision-making but also when deciding whether a genetic technology is morally good or morally bad, we decided to use different questionnaire, as every questionnaire focuses on a slightly different topic. Furthermore, the participants were asked about their attitudes toward the availability of genetic tests, and a comparison was made between the two countries.

The GKGH failed to achieve the desired properties in internal consistency and item analysis within the German sample. It faced a significant ceiling effect, but the German group displayed good values in the retest and construct validity. In contrast, the Greek group excelled in all aspects and did not encounter the issues observed in the German group. PCA revealed a similar three-factor structure in both samples, with one item in both groups not aligning with any factor.

The KGEI demonstrated adequate internal consistency in both groups, favorable values in item analysis, and performed well in the known-groups method. However, in the retest, the German group exhibited low values, whereas the Greek group maintained satisfactory values. Notably, the German group showed a significant ceiling effect that was absent in the Greek group. PCA identified an identical two-factor structure in both groups, with a few items not falling under any factor.

Furthermore, the KMGG consistently exhibited positive properties across all categories in both samples, although the Greek group experienced a notable floor effect. The German sample revealed a three-factor structure for structural validity, while the Greek group lacked a coherent structure.

Across all three questionnaires, participants with higher education levels tended to achieve higher scores. Results for the influence of age and gender were mixed, with no discernible differences based on religion in any of the questionnaires.

In the Greek group, prior experiences with genetic testing were associated with higher knowledge scores in the questionnaires, unlike in the German sample. Regarding preferences for the availability of genetic tests, both countries generally yielded comparable results. The sale of tests online was critically scrutinized by both groups, with a preference for genetic testing through medical prescriptions evident in both.

### Internal consistency and retest

The questionnaire “General Knowledge of Genes and Heredity” was originally developed by Jallinoja and Aro (1999). It was subsequently used to examine genetic knowledge in the general population (Jallinoja and Aro, 2000; Haga et al., 2013) and in patients with chronic diseases (Calsbeek et al., 2007) as well as to evaluate the difference between healthy people and patients with immune diseases (Khdair et al., 2021).

Although the initial publication exhibited a Cronbach’s alpha of 0.86, the German adaptation yielded a lower score of 0.646. Notably, the Cronbach’s alpha for the subset of individuals who perceive themselves as highly knowledgeable about genetic technologies was even lower at 0.446.

However, the decline in internal consistency was not evident in the Greek group. This is likely due to the questionnaire being relatively easy for the German group, as eight items were answered almost completely correctly. This fact led to a significant reduction in the variance among the items, as there was little variability. Consequently, the other psychometric properties in the German group did not perform very well, while the questionnaire in the Greek group consistently demonstrated good properties.

When compared to other studies, the Greek group’s rate of correctly answered items was similar to that of various samples, including the Finnish group (63.5% correct answers), as observed by Jallinoja and Aro in 1999, the Dutch group (45.8%) in the study by Calsbeek et al. (Calsbeek et al., 2007), the Jordanian group (65.4%) in the research by Khdair et al. (2021), and the Chinese group (59.2%), according to Zhang et al. (Zhang et al., 2020). In contrast, the German group scored notably higher at 82.8%. Nevertheless, it is strikingly similar to a U.S. sample reported by Haga et al. (2013), which achieved 83.6% correct answers. Those studies that reported Cronbach’s alpha values all had less than 70% correct answers but exhibited internal consistencies of over 0.8. This suggests that the questionnaire’s internal consistency depends on the composition of the sample.

The KGEI and KMGG questionnaires, developed by Carver et al. (2017), are relatively recent instruments primarily utilized in studies investigating beliefs related to genetic determinism within the context of genetic knowledge (Gericke et al., 2017). Initially, Carver et al. (2017) suggested treating both knowledge questionnaires as a single scale. However, Tornabene et al. (2020) challenged this idea, demonstrating in a PCA that these questionnaires should be considered as two separate tools. As a result, we recognized them as two independent instruments for assessing knowledge.

In comparison to other validation studies, our sample results were notably strong. While Carver et al. (2017), Tornabene et al. (2020), and Gericke et al. (2017) reported internal consistency values ranging from 0.61 to 0.69 for the KGEI and KMGG, we observed substantially higher values. Similarly, the Spanish translation by Subasic et al. (2021) reported 0.782 for the KGEI and 0.926 for the KMGG, although it is important to acknowledge that Subasic’s sample was relatively small, consisting of only 20 participants.

Variations in internal consistency are often linked to the characteristics of the sample used. For instance, Gericke et al. (2017) exclusively recruited first-year Brazilian undergraduates from one university, Carver et al. (2017) also recruited only first-year students from the same university, and Tornabene et al. (2020) included students solely from an introductory biology class. Consequently, it is not surprising that our samples exhibited different properties.

The test-retest reliability was good for the GKGH and KMGG but did not meet the desired standards for the KGEI. It should be noted that the reduction in the sample size of the retest groups can lead to a degradation of the reliability assessment (Polit, 2014; Aldridge et al., 2017), resulting in an underestimation.

### Construct validity

The construct validity of all three knowledge questionnaires was confirmed through the known-groups method, as they were able to differentiate between groups with low and high self-assessed knowledge of biotechnology. In future validations, it is advisable to use different measures, as self-assessment is not an objective evaluation.

### Item analysis and floor and ceiling effects

The GKGH displays varying item analysis characteristics depending on the sample. The weak inter-item correlation within the German group can be attributed to the fact that some of the items received nearly perfect answers, leaving little room for correlations. This result is reinforced by the observed ceiling effect, suggesting that the questionnaire is too easy for this particular sample. Accordingly, these issues were not observed in the Greek group, which, overall, has lower knowledge of gene technologies and the results for the item analysis were satisfactory for the Greek sample. Unfortunately, there are no comparable studies that have conducted item analyses.

During the item analysis of the KGEI, a notable observation emerged, consistent with the observation made by Tornabene et al. (2020), that the questionnaire is relatively easy and better suited for samples with comparatively lower knowledge of genetic technologies. We were able to substantiate this in our two groups: The German group, characterized by higher knowledge, experienced a significant ceiling effect, while the Greek group, with lower knowledge, consistently performed well in the item analysis without encountering any issues.

The KMGG presents a contrasting issue compared to the GKGH. It is well-suited for the German sample but proves to be too demanding for the Greek group, as indicated by a significant floor effect. When examining other samples where the KGEI and KMGG have been employed, it becomes apparent that the KGEI tends to have a moderate level of difficulty, while the KMGG appears relatively challenging (Carver et al., 2017; Gericke et al., 2017; Tornabene et al., 2020; Subasic et al., 2021), which aligns with the findings in our samples. Carver et al. (2017) reported that the difficulty of the KMGG was associated with items related to epigenetics, which posed significant challenges for participants but were retained to reflect the current state of research. However, in our samples, we did not observe a similar pattern, as the difficulty of the items appeared relatively balanced across various topics.

Beyond the questionnaires’ performance, we would like to underscore a crucial critique, as previously noted by Tornabene et al. (2020) and evident from the factor loadings of the items: The KGEI does not measure the construct implied by its title. It lacks items related to gene-environment interactions, focusing solely on the influence of genes on traits and diseases as well as the influence of the environment. To effectively assess knowledge about gene-environment interactions, the questionnaire would need to be expanded with additional items that specifically address this aspect.

### Socioeconomic influences

#### Education

The connection between the GKGH score and education has been extensively documented in previous studies (Jallinoja and Aro, 1999; Calsbeek et al., 2007; Haga et al., 2013). Our research also supports this relationship, as we have found evidence that a higher level of education is associated with a greater knowledge of genetic technologies. However, Dar-Nimrod et al. (2018) did not observe this correlation in their sample, suggesting that higher education does not necessarily indicate an individual’s engagement with the topic of genetics or epigenetics within their education. The KGEI and KMGG exhibit a similar relation, but due to their limited usage, there is a lack of comparable studies. Nevertheless, a positive trend is evident in our samples, implying that a more extensive education is associated with a greater knowledge of genetics.

Nevertheless, it is important to approach the education variable with caution, as we only distinguished between academic and non-academic qualifications. It would be beneficial in the future to utilize more precise measures in this regard.

#### Gender

No significant correlation was identified between gender and the overall score, and only the Greek sample showed significant differences between women and men. This finding aligns with previous studies on the GKGH, where gender was not considered a significant factor (Jallinoja and Aro, 1999; Calsbeek et al., 2007; Zhang et al., 2020). Comparable studies for the KGEI and KMGG are therefore still needed. Gender differences in genetic knowledge have been observed in large international samples, where it was also noticeable that men tend to have slightly higher levels of knowledge (Chapman et al., 2019).

#### Age

The impact of age on knowledge yields mixed results. In our study, we found no such influence in the case of the GKGH, which aligns with the findings of Zhang et al. (2020) and Khdair et al. (2021). However, Haga et al. (2013), Calsbeek et al. (2007), and Jallinoja and Aro (1999) reported a negative correlation between age and overall scores. We only detected this negative correlation between knowledge scores and age in the total sample for the KGEI and KMGG. We also identified a significant difference between younger and older participants in the case of the KGEI. Therefore, it is reasonable to conclude that age does not exert a consistent influence on the knowledge scores, although it is crucial to emphasize that the categorization of participants’ ages varies significantly.

Little et al. (2022) suggested in their research that knowledge about biotechnology has generally increased in the United States over the past decade. They attributed this trend, in part, to the availability of information and media presence. However, according to previous PISA reports, which assess the academic performance of OECD countries at regular intervals, there has been a steady decline in overall academic performance in the natural sciences since 2006 (OECD, 2019; OECD, 2023). Hence, it is plausible to assume that age plays a more intricate role than previously thought, and cohort effects might need to be taken into consideration.

#### Religion

Regarding the influence of religiosity, there is a scarcity of studies on this topic. In the total sample, we observed a weak negative correlation between the GKGH score and religiosity, a relationship that Dar-Nimrod et al. (2018) did not observe. However, their study utilized only specific items from the questionnaire rather than the entire questionnaire. In the case of the KGEI and KMGG, Gericke et al. (2017) illustrated in their research that students who declared strong religious influence achieved notably lower scores in the KGEI. Nonetheless, our group tests, encompassing individuals with varying levels of religiosity, did not reveal such distinctions in any of the three questionnaires.

At present, it is speculated that religiosity may act as a moderating factor in the link between knowledge of scientific content and attitudes toward genetic interventions (Allum et al., 2014) rather than having a direct impact on them. However, Chapman et al. (2019) also demonstrated that the type of religion itself exerts influence on genetic knowledge. Since this project did not inquire about the attitudes of the participants or specifics about their religious beliefs, this area presents the potential for future research.

### Comparison between Germany and Greece

When comparing the two countries, the most noteworthy difference lies in the average scores of the two sample groups in the knowledge questionnaires. A key factor in this disparity is the variation in the educational systems of the two countries. In Greece, it is possible to complete one’s education without taking a natural science course in the upper grades, resulting in lower knowledge about biology.

The previously mentioned PISA study vividly underscores the gap between Germany and Greece: Germany scored 503; SD = 103 (2019) and 492; SD = 106 (2022) in natural sciences, while Greece scored 452; SD = 86 and 441; SD = 91 (2022) (OECD, 2019; OECD, 2023). The score difference was deemed statistically significant for both years. Moreover, as per PISA 2019 data, which is the most recent available data for gender differences, Greece demonstrates some of the most substantial gender disparities among all OECD countries, a trend we observed in the group differences within our tests. In contrast, gender differences in Germany in natural sciences are not statistically significant according to PISA, which we could not detect for any of the three questionnaires. It is worth noting that PISA primarily assesses general knowledge in natural sciences for approximately 15-year-old students. However, in essence, it is evident that there is a need for improvement, as has been indicated in numerous other studies, as genetic knowledge seems to be low, even among the well-educated (Chapman et al., 2019).

When comparing Germany and Greece in the questionnaire on potential providers of genetic testing questionnaire, there are barely any differences between the two countries, and the two samples are distributed very similarly. It becomes evident that age, gender, and religiosity are all factors that play a role in attitudes toward the availability of genetic tests. However, there is no distinction between academic and non-academic participants when asked about the access to genetic tests.

In comparison with Chokoshvili’s study (Chokoshvili et al., 2017), which analyzed a Belgian sample, our groups appear significantly more open to the availability of genetic tests. For instance, the sale of genetic tests through the Internet was almost universally denied in Chokoshvili’s sample, while our DE and GR groups, although displaying a negative trend, had some participants respond with neutral or even a positive attitude.

Furthermore, we could identify the same socioeconomic differences for age, gender, and religion but not education. Chokoshvili et al. (2017) have reported some differences regarding the education of the participants, which we did not observe in either of our two samples, noting that they have chosen a slightly different categorization for the education variable, which, in any case, should be examined with more granularity. However, when it came to the question of marketability via the internet, these differences were not significantly detectable, as the question received unanimous denials. At the same time, we did manage to observe differences for gender, age, and religion.

Moreover, we made another interesting observation regarding prior experiences with genetic testing: In the German sample, it made no difference whether an individual had previously undergone a genetic test or had a friend or acquaintance who had taken a test, or expressed a desire to have one. In contrast, within the Greek group, participants who had undergone a test themselves achieved significantly higher knowledge scores in two of the questionnaires. Similar patterns were observed among those who were familiar with someone in their social network who had participated in a genetic test. Accordingly, this suggests that the utilization of genetic testing services appears to depend on more factors than previous experience with genetic testing and should be considered further in the future.

In summary, it is important to note that none of the three questionnaires managed to impress consistently. Nevertheless, we believe that all three questionnaires have the potential to yield valid results if the knowledge level of the sample is taken into consideration. The GKGH and KGEI appear to be effective in samples with a relatively lower understanding of genetics, achieving the desired psychometric properties, while the KMGG performed exceptionally well in samples with a high level of knowledge. It is worth mentioning that the three questionnaires cover slightly different subject areas, so careful evaluation of the content is necessary when selecting the measurement tool, particularly with the assertion of the KGEI, which claims to assess knowledge about gene-environment interactions.

### Strengths and limitations

Although we made efforts to ensure a diverse sample by including individuals from various social groups and encouraging their social circles to participate in the study, upon comparing our sociodemographic data to the general population in Germany and Greece, it appears that our sample is likely to have a higher level of education (Federal Statistical Office Germany, 2019; Statista, 2022).

In the current German population, 33% of individuals aged 25 to 64 hold a university degree. In Greece, this percentage is 34% for the same age group, while in the Eurozone as a whole, it stands at 38%, with a steady increase observed since 2014 (Federal Ministry of Education and Research Germany, 2022).

Considering that the average age in our sample is 43 and 41 years, respectively, comparing to available statistics for individuals aged 30–34 provides insightful cohort differences and population trends. In the Eurozone, 43.2% of individuals aged 30 to 34 now hold a university degree. In Greece, the figure is 45.2%, and in Germany, it is 37.1% (Statista, 2022). Notably, over the past decade, the proportion of women with university degrees within a generation in Germany has doubled, and the overall percentage of university graduates continues to rise (Federal Ministry of Education and Research Germany, 2018).

However, it is important to note that our sample demonstrates a higher educational attainment compared to the general population, which indicates a potential bias that should be acknowledged.

This bias is a known issue, possibly stemming from the lower participation of less educated individuals in scientific projects. Additionally, the recruitment channels we utilized primarily targeted individuals with a specific interest in research projects. It is also important to mention that those participants who take part again after a 4-week interval probably have a higher interest in the topic of genetics, which could lead to a potential bias.

Besides, it is important to consider the limitations associated with the online survey format. Online surveys tend to attract respondents who are technologically proficient or have ample free time (Wright, 2005; Hunter, 2012), leading to a potential selection bias and skewed results. Moreover, the absence of personal interaction, as seen in face-to-face interviews, restricts the ability to delve into more detailed or nuanced responses (Ball, 2019). Lastly, technical difficulties like slow loading times or issues with the survey software can frustrate respondents and potentially impact response rates.

Therefore, for future projects, we recommend larger sample sizes and propose an expanded recruitment program. Convenience samples, in general, tend to be statistically biased due to their composition being predominantly WEIRD (Western, Educated, Industrialized, Rich, and Democratic) (Henrich et al., 2010). Thus, the ability to generalize and make cross-cultural comparisons is limited.

One inherent issue in this study is the potential for respondent fatigue resulting from completing many consecutive scales. The order of these scales was not randomized, since our survey instrument does not support this method. Additionally, the knowledge about genetic technologies was arbitrarily assessed in this study and should instead be measured using a validated questionnaire in future projects.

## Conclusions

The GKGH and KMGG demonstrate robust psychometric properties, serving as valid tools for gauging knowledge of genetic engineering. They cover slightly varied subjects, with KMGG incorporating newer knowledge elements on epigenetics. For samples with lower expected knowledge of genetic technologies, we recommend utilizing the GKGH, given its overall greater ease compared to the KMGG. Despite acceptable values, the KGEI lacks validity as a questionnaire for its title, as it does not include items on gene-environment interaction and overall presents a weaker performance in knowledge assessment compared to the other two. The German group consistently outperformed the Greek group on all questionnaires, highlighting the significant role of education. Gender, religiosity, and participant age yield diverse results, offering intriguing avenues for future research. Preferences for the availability of genetic tests are alike in both countries, generally opposing online sales, yet opinions differ based on age, religious beliefs, and gender.

## Scope statement

Our research contributes to the expanding field of biosciences, focusing on recent advancements in genetic technologies. While genetic tests and gene-editing tools have revolutionized medicine and technology, there is a notable absence of reliable instruments to assess public knowledge about these technologies. In response, our study aimed to fill this gap by translating and validating scales to effectively measure genetic knowledge in the general population for the German and Greek languages. For this purpose, three previously developed instruments were compared through statistical procedures, and their quality was evaluated, enabling recommendations for future research. Additionally, we compare the German and Greek samples, examining whether sociodemographic differences such as age, gender, education, and religiosity impact knowledge about genetic technologies and what preferences the two countries have regarding the availability of genetic tests.

## Data Availability

The raw data supporting the conclusions of this article will be made available by the authors, without undue reservation.

## Supplementary material

Supplementary Table 1 STROBE-Checklist.

Supplementary Table 2 Translation of the GKGH, KGEI and KMGG in Greek and German.
