# Supplementary material for: Translation, validation, and comparison of genetic knowledge scales in Greek and German
Source: Front Genet. 2024 May 15;15:1350308. doi: 10.3389/fgene.2024.1350308 (PMC11133520; doi:10.3389/fgene.2024.1350308)
Supplement: Supplementary file 2 [file Table2.docx]

**General Knowledge of Genes and Heredity (Jallineja & Ano 1999)**

|  | **EN** | **DE** | **GR** |
| --- | --- | --- | --- |
| 1 | One can see a gene with the naked eye. | Man kann ein Gen mit dem bloßen Auge sehen. | Μπορείτε να δείτε ένα γονίδιο με γυμνό μάτι. |
| 2 | A gene is a disease. | Ein Gen ist eine Krankheit. | Ένα γονίδιο είναι μια ασθένεια. |
| **3 [a]** | **A gene is a piece of DNA.** | **Ein Gen ist ein Abschnitt innerhalb der DNA, der die Bauanleitung für ein Protein enthält.** | **Το γονίδιο είναι ένα μέρος του DNA που περιλαμβάνει τις οδηγίες κατασκευής μίας πρωτεΐνης.** |
| **4** | **Genes are inside cells.** | **Gene befinden sich innerhalb der Zellen.** | **Τα γονίδια βρίσκονται στο εσωτερικό των κυττάρων.** |
| 5 | A gene is a cell. | Ein Gen ist eine Zelle. | Ένα γονίδιο είναι ένα κύτταρο. |
| **6** | **A gene is a part of a chromosome.** | **Ein Gen ist ein Teil eines Chromosoms.** | **Ένα γονίδιο είναι ένα τμήμα ενός χρωμοσώματος.** |
| 7 | Different body parts include different genes. | Verschiedene Körperteile enthalten verschiedene Gene. | Διαφορετικά μέρη του σώματος περιέχουν διαφορετικά γονίδια. |
| 8 | Genes are bigger than chromosomes. | Gene sind größer als Chromosomen. | Τα γονίδια είναι μεγαλύτερα από τα χρωμοσώματα. |
| **9** | **The genotype is not susceptible to human intervention.** | **Der Genotyp ist nicht zugänglich für menschliche Eingriffe.** | **Ο γονότυπος δεν είναι προσβάσιμος στην ανθρώπινη παρέμβαση.** |
| **10 [a]** | **It has been estimated that a person has about 70,000 genes.** | **Eine Person hat schätzungsweise 20000 Gene.** | **Ένα άτομο έχει περίπου 20.000 γονίδια.** |
| **11** | **Healthy parents can have a child with a hereditary disease.** | **Gesunde Eltern können ein Kind mit einer Erbkrankheit bekommen.** | **Οι υγιείς γονείς μπορούν να αποκτήσουν παιδί με κληρονομική ασθένεια.** |
| **12** | **The onset of certain diseases is due to genes, environment and lifestyle.** | **Das Auftreten  bestimmter Krankheiten ist auf die Gene, die Umwelt und den Lebensstil zurückzuführen.** | **Η εμφάνιση ορισμένων ασθενειών μπορεί να οφείλεται στα γονίδια, το περιβάλλον και τον τρόπο ζωής.** |
| **13** | **The carrier of a disease gene may be completely healthy.** | **Der Träger eines Krankheitsgens kann völlig gesund sein.** | **Ο φορέας ενός γονιδίου ασθένειας μπορεί να είναι απολύτως υγιής.** |
| 14 | All serious diseases are hereditary. | Alle schweren Krankheiten sind erblich bedingt. | Όλες οι σοβαρές ασθένειες είναι κληρονομικές. |
| 15 | The child of a disease gene carrier is always also a carrier of the same disease gene. | Das Kind eines Krankheitsgenträgers ist immer auch Träger desselben Krankheitsgens. | Το παιδί ενός φορέα γονιδίου ασθένειας είναι πάντα επίσης φορέας του ίδιου γονιδίου ασθένειας. |

Notes: [a] the item was adapted. Bold statements are correct

**Knowledge about Gene-Environment Interaction (Carver et al., 2017)**

|  | **EN** | **DE** | **GR** |
| --- | --- | --- | --- |
| 1 | A gene codes directly for a trait or disease. | Ein Gen codiert direkt für ein Merkmal oder eine Krankheit. | Ένα γονίδιο κωδικοποιεί άμεσα ένα χαρακτηριστικό ή μια ασθένεια. |
| 2 | Most human traits and diseases are caused by a single gene. | Die meisten menschlichen Merkmale und Krankheiten werden durch ein einziges Gen verursacht. | Τα περισσότερα ανθρώπινα χαρακτηριστικά και ασθένειες προκαλούνται από ένα μόνο γονίδιο. |
| **3** | **A single gene can influence several different traits or diseases.** | **Ein einzelnes Gen kann mehrere verschiedene Merkmale oder Krankheiten beeinflussen.** | **Ένα και μόνο γονίδιο μπορεί να επηρεάσει πολλά διαφορετικά χαρακτηριστικά ή ασθένειες.** |
| 4 | A person´s height is influenced by one gene only. | Die Körpergröße eines Menschen wird durch ein einziges Gen beeinflusst. | Το ύψος ενός ατόμου επηρεάζεται από ένα μόνο γονίδιο. |
| **5** | **Most traits and diseases are influenced by many different genes.** | **Die meisten Merkmale und Krankheiten werden von vielen verschiedenen Genen beeinflusst.** | **Τα περισσότερα χαρακτηριστικά και ασθένειες επηρεάζονται από πολλά διαφορετικά γονίδια.** |
| 6 | Most traits and diseases are caused by environmental factors only (such as diet and lifestyle). | Die meisten Merkmale und Krankheiten werden ausschließlich durch Umweltfaktoren (wie Ernährung und Lebensstil) verursacht. | Τα περισσότερα χαρακτηριστικά και ασθένειες προκαλούνται αποκλειστικά από περιβαλλοντικούς παράγοντες (όπως η διατροφή και ο τρόπος ζωής). |
| 7 | A gene can only influence a single trait or disease. | Ein Gen kann nur eine einzige Eigenschaft oder Krankheit beeinflussen. | Ένα γονίδιο μπορεί να επηρεάσει μόνο ένα χαρακτηριστικό ή μια ασθένεια. |
| **8** | **Most traits and diseases are caused by both genes and environmental factors.** | **Die meisten Merkmale und Krankheiten werden sowohl von Genen als auch von Umweltfaktoren verursacht.** | **Τα περισσότερα χαρακτηριστικά και ασθένειες προκαλούνται τόσο από γονίδια όσο και από περιβαλλοντικούς παράγοντες.** |
| **9** | **A person´s height is influenced by many different genes.** | **Die Körpergröße einer Person wird von vielen verschiedenen Genen beeinflusst.** | **Το ύψος ενός ατόμου επηρεάζεται από πολλά διαφορετικά γονίδια.** |

Notes: Bold statements are correct.

**Knowledge about Modern Genetics and Genomics (Carver et al., 2017)**

|  | **EN** | **DE** | **GR** |
| --- | --- | --- | --- |
| 1 | The genome consists only of the genes in an organism that code for the production of proteins. | Das Genom besteht nur aus den Genen in einem Organismus, die für die Produktion von Proteinen codieren. | Το γονιδίωμα αποτελείται μόνο από τα γονίδια ενός οργανισμού που κωδικοποιούν την παραγωγή πρωτεϊνών. |
| **2** | **Cells, tissues and organs differ because they have different sets of genes that are activated (“turned on”) and deactivated (“turned off”).** | **Zellen, Gewebe und Organe unterscheiden sich voneinander, weil sie unterschiedliche Sätze von Genen haben, die aktiviert ("eingeschaltet") und deaktiviert ("ausgeschaltet") werden.** | **Τα κύτταρα, οι ιστοί και τα όργανα διαφέρουν μεταξύ τους επειδή διαθέτουν διαφορετικά σύνολα γονιδίων που ενεργοποιούνται και απενεργοποιούνται.** |
| 3 | Environmental factors, such as cigarette smoke, can affect gene activity. | Umweltfaktoren wie Zigarettenrauch können die Genaktivität beeinflussen. | Περιβαλλοντικοί παράγοντες, όπως ο καπνός του τσιγάρου, μπορούν να επηρεάσουν τη γονιδιακή δραστηριότητα. |
| 4 | The human genome contains more genes than the genome of any other living being. | Das menschliche Genom enthält mehr Gene als das Genom eines jeden anderen Lebewesens. | Το ανθρώπινο γονιδίωμα περιέχει περισσότερα γονίδια από το γονιδίωμα οποιουδήποτε άλλου έμβιου όντος. |
| **5** | **Every cell of the body contains the whole genome.** | **Jede Zelle des Körpers enthält das gesamte Genom.** | **Κάθε κύτταρο του σώματος περιέχει ολόκληρο το γονιδίωμα.** |
| **6** | **When someone says something is “epigenetic” it means that you can inherit changes in gene activity without inheriting changes in the genes.** | **Wenn jemand sagt, dass etwas "epigenetisch" ist, bedeutet das, dass man Veränderungen in der Genaktivität vererben kann, ohne Veränderungen in den Genen zu erben.** | **Όταν κάποιος λέει ότι κάτι είναι "επιγενετικό", σημαίνει ότι μπορεί να κληρονομηθούν αλλαγές στη γονιδιακή δραστηριότητα χωρίς να κληρονομηθούν αλλαγές στα γονίδια.** |
| **7** | **Only a small proportion of the human genome consists of genes that code for proteins.** | **Nur ein kleiner Teil des menschlichen Genoms besteht aus Genen, die für Proteine kodieren.** | **Μόνο ένα μικρό μέρος του ανθρώπινου γονιδιώματος αποτελείται από γονίδια που κωδικοποιούν πρωτεΐνες.** |
| 8 | Epigenetic changes are influenced by environmental factors. | Epigenetische Veränderungen werden durch Umweltfaktoren beeinflusst. | Οι επιγενετικές αλλαγές επηρεάζονται από περιβαλλοντικούς παράγοντες. |
| **9** | **When someone talks of an epigenetic change he or she is referring to a large change in the DNA sequence.** | **Wenn jemand von einer epigenetischen Veränderung spricht, meint er oder sie eine große Veränderung in der DNA-Sequenz.** | **Όταν κάποιος μιλάει για επιγενετική αλλαγή, εννοεί μια σημαντική αλλαγή στην αλληλουχία του DNA.** |
| 10 | Epigenetic changes are caused by mutations. | Epigenetische Veränderungen werden durch Mutationen verursacht. | Οι επιγενετικές αλλαγές προκαλούνται από μεταλλάξεις. |
| 11 | Most of the human genome consists of genes that code for proteins. | Der größte Teil des menschlichen Genoms besteht aus Genen, die für Proteine kodieren. | Το μεγαλύτερο μέρος του ανθρώπινου γονιδιώματος αποτελείται από γονίδια που κωδικοποιούν πρωτεΐνες. |
| **12** | **The human genome has fewer genes than some less complex organisms such as tomato plants and rice.** | **Das menschliche Genom hat weniger Gene als einige weniger komplexe Organismen wie Tomatenpflanzen und Reis.** | **Το ανθρώπινο γονιδίωμα έχει λιγότερα γονίδια από ορισμένους λιγότερο πολύπλοκους οργανισμούς, όπως τα φυτά ντομάτας και το ρύζι.** |
| 13 | When someone says something is “epigenetic”, it means that environmental factors can change part of the DNA sequence | Wenn jemand sagt, dass etwas "epigenetisch" ist, bedeutet das, dass Umweltfaktoren einen Teil der DNA-Sequenz verändern können. | Όταν κάποιος λέει ότι κάτι είναι "επιγενετικό", σημαίνει ότι περιβαλλοντικοί παράγοντες μπορούν να αλλάξουν μέρος της αλληλουχίας του DNA. |
| **14** | **Genes can be activated or deactivated by other genes.** | **Gene können von anderen Genen aktiviert oder deaktiviert werden.** | **Τα γονίδια μπορούν να ενεργοποιηθούν ή να απενεργοποιηθούν από άλλα γονίδια.** |
| 15 | Only eye cells have genetic information for eye colour. | Nur Augenzellen haben die genetische Information für die Augenfarbe. | Μόνο τα κύτταρα των ματιών έχουν τις γενετικές πληροφορίες για το χρώμα των ματιών. |
| **16** | **If a cell lacks a certain substance, such as a vitamin, a gene can be deactivated.** | **Wenn einer Zelle eine bestimmte Substanz fehlt, z. B. ein Vitamin, kann ein Gen deaktiviert werden.** | **Εάν ένα κύτταρο στερείται μιας συγκεκριμένης ουσίας, π.χ. μιας βιταμίνης, ένα γονίδιο μπορεί να απενεργοποιηθεί.** |

Notes: Bold statements are correct.
